# Effect of locosamide as an add-on therapy in the management of dissociative disorders

**DOI:** 10.6026/973206300200373

**Published:** 2024-04-30

**Authors:** Sambhu Prasad, Anant Kumar Verma, Santosh Kumar, Sweta Gupta

**Affiliations:** 1 Department of Psychiatry, AIIMS, Patna, Bihar, India; 2 Department of Psychiatry, Bhagwan Mahavir Institute of Medical Science, Pawapuri, Nalanda, Bihar, India; 3 Department of Psychiatry, IGIMS, Patna, Bihar, India; 4 Department of Orthodontics and Dentofacial Orthopaedics, Patna Dental College and Hospital, Patna, Bihar, India

**Keywords:** Lacosamide, dissociative disorders, randomised control trial

## Abstract

The role of lacosamide (LCM) as add on treatment modality in dissociative disorders (DD) is of interest. It was a randomized control trial in which 300 patients diagnosed with dissociative disorders having treatment for the dissociative disorders were included. They were divided into two groups. Group one consisted of intervention group in which LCM was also administered along with conventional psychiatric medication for different dissociative disorders. Group two consisted of control group where the patients of dissociative disorders were found to have conventional medication. There was analysis of improvements in recovery of symptoms and quality of life. There was statistically significant increase in excellent, very good, good and fair quality of life and decrease in poor and satisfactory quality of life in intervention group after drug intervention. It was observed that symptoms of the patients improved in 50.67% cases in intervention group and 10.67% cases in control group. There was greater improvement in recovery of symptoms and quality of life in patients of DD in which LCM was administered as add on medication.

## Background:

Mental health illnesses known as dissociation disorders (DDs) are characterized by a lack of relationship with one's identity, environment, ideas, recollections, and emotions [1, 2, 3]. Among these situations is the unwelcome and unhealthy escape from reality. This makes day-to-day living difficult [5, 6]. Dissociative disorders typically develop in response to upsetting, painful, or disturbing experiences and aid in erasing unpleasant memories [7, 8, 9]. Dissociative disorder types can have a variety of symptoms, from diminished memory to dissociated identities. Stressful times can temporarily exacerbate symptoms and make them more noticeable [10, 11, 12]. Talk therapy, commonly known as psychotherapy, and medication are two possible treatments for DDs [13, 14, 15]. Dissociation may involve an increase in self-control and arousal modulation ("emotion overmodulation"), associated with increased activation in frontal regions (dorsal/rostral anterior cingulate cortex (ACC), medial prefrontal cortex (mPFC)) and dampened limbic activity in the amygdala and insula [16, 17, 18, 19]. Patients predominantly showing hyperarousal and re-experiencing symptoms (emotion under-modulation) are thought to exhibit the reverse pattern: limbic hyperactivity and diminished recruitment of the ACC and mPFC. Central to this model were observations from a script-driven imagery study, in which post-traumatic stress disorder (PTSD) patients were exposed to autobiographical narratives of traumatic events [20, 21, 22]. The majority of patients reported pronounced re-experiencing symptoms (traumatic flashbacks, intense feelings of shame, disgust, guilt, hopelessness, etc.), associated with an increase in heart rate [13- 18]. In contrast, patients with the dissociative subtype demonstrated increased activity in areas implicated in emotion regulation, arousal modulation, and sensory filtering (e.g., medial frontal gyrus, anterior cingulate, middle temporal gyri, precuneus, occipital areas, and inferior frontal gyrus) [19-23]. Patients with the dissociative subtype exhibited a stronger coupling of the ventrolateral thalamus with right insula, middle frontal gyrus, superior temporal gyrus, cuneus, and left parietal lobe (regions implicated in emotion regulation), while connectivity between the thalamus and right parahippocampalgyrus and superior occipital gyrus (areas implicated in sensory and emotion processing) was diminished [18-24]. Interestingly, patients with the dissociative subtype exhibited enhanced activity in the ventral PFC, suggesting increased emotion downregulation, when threatening (fearful versus neutral) facial expressions were presented on a conscious level-but not when threatening stimuli were presented non-consciously. In the latter condition, these patients showed increased activity in the amygdala, insula, and thalamus, suggesting that regulatory processes during dissociation may be partly explained by conscious top-down processes, which might not work on a non-conscious level [4-8]. For many years, anticonvulsant drugs have been utilized to treat mental health issues. It is hypothesised that the molecular pathways via which they prevent seizures may also result in behavioral and emotional stabilization [9-12]. Valproate, carbamazepine, and lamotrigine have all shown promise in treating acute mania in adults and preventing recurrence of bipolar disease when used as maintenance medication [15-18]. While topiramate and oxycarbazepine are also utilized, their effectiveness has not been proven. Furthermore, it has been observed that several anticonvulsants possess anti-aggressive characteristics, and that phenytoin, carbamazepine, and oxcarbazepine are efficacious in treating recurring episodes of impulsive aggression [19-24]. While managing DDs can be challenging, many patients find improved quality of life and new coping mechanisms. For those suffering from epilepsy, antiseizure medicines (ASMs) are crucial in managing psychological comorbidities [15, 16]. While some ASMs-like mood stabilizers-have been shown to reduce psychological symptoms, others may have mental adverse effects of their own [16-18]. A newer ASM called lacosamide (LCM) preferentially improves gradual sodium-channel inactivation [17- 20]. It is recommended as supplementary therapy for individuals with seizures that have focal onset and as monotherapy at dosages up to 400 mg/day in Japan. LCM can help adult epileptic patients regulate their seizures and is often well tolerated by those with mental health issues and cognitive impairments [18- 21]. The implications of LCM on psychological comorbidities, however, have not received much attention [12-16]. One study found that LCM was both well-tolerated and efficacious in individuals with epilepsy who also had psychiatric complications, the most common of which were anxiety and sadness [19-21]. Systematic studies on the impact of LCM on individuals with severe psychological comorbidities, like psychosis as well as irritability, are lacking, though. Therefore, it is of interest to evaluate the role of LCM as add on treatment modality in dissociative disorders.

## Materials and Methods:

It was a randomised control trial in which 300 patients diagnosed with dissociative disorders having treatment for the dissociative disorders were included. They were divided into two groups. Group one consisted of intervention group in which LCM was also administered along with conventional psychiatric medication for different dissociative disorders. Group two consisted of control group where the patients of dissociative disorders were found to have conventional medication.

Group A= Intervention group (n=150)

Group B= Control group (n=150)

Patients who were monitored for at least three months following LCM treatment and were being followed as outpatients were the ones we enrolled. The administration of LCM involved a progressive increase of 100 mg over a minimum of 4 weeks, beginning at an initial dose of 100 mg per day. There was analysis of improvements in recovery of symptoms and quality of life. In dissociative disorder we used to cut down secondary gain as treatment.For three reasons, it was not appropriate to reduce secondary gains too early in the course of treatment and without providing the family with sufficient justifications. First, the source of the symptoms may not be known to the doctor. Second, a decrease in secondary gain could be interpreted by the family as patent neglect. Moreover, at first, the family might not have complete faith in the doctor and the hospital to take care of their patient.

## Improvement in symptoms recovery and quality of life:

Information from healthcare records was used to assess how LCM polytherapy affected modifications to psychiatric signs and symptoms. Patients were also categorized into "improved," group "unchanged," group and "worsened" groups based on psychiatric symptoms. The evaluation of psychiatric symptoms was based on the complaints made by individuals or assessments made by their relatives or caretakers. We also looked into which ASMs were started or stopped in each patient following the administration of LCM. The World Health Organization (WHO) has developed a quality of life instrument, the WHOQOL, which captures many subjective aspects of quality of life). The WHOQOL-BREF is one of the best known instruments that has been developed for cross-cultural comparisons of quality of life and is available in more than 40 languages [26].An abbreviated version of the WHOQOL-BREF that contains 26 items is applicable in clinical trials in which brief measures are needed, and also in epidemiological studies in which quality of life might be one of several outcome variables. The WHOQOL BREF covers four different domains of quality of life [27]. The WHOQOL is under cross-cultural validation by the WHOQOL group. Quality of life (QoL) changes were categorized as "Excellent," "Very Good," "Good," "Fair," "Satisfactory," and "Poor."

## Statistics:

IBM SPSS Statistics 24 was used to conduct the statistical analysis (IBM Corp., Armonk, NY, US). To determine if alterations in psychological signs were connected to the likelihood of seizures, we employed the chi-squared test. A significance level of p < 0.05 was used.

## Results:

The mean age of study participants in study group and control group was 34.54±1.34 years and 36.23± 1.21 years respectively. The proportion of females was greater in both study group and control group. The different diagnosis of dissociative disorders in study group were irritability (36.00%), psychosis (27.34%), anxiety (11.34%), amnesia (4.67), personality change (4.67%), obsessive compulsive disorders (2.67%), depression (2.67%) and psychogenic non epileptic seizures (10.67%). The proportion of different dissociative disorders was comparable in control group (p=0.965) (Table 1). The quality of life was comparable in study participants in both control group and intervention group before the intervention of locosamide (p=0.791) (Table 2). There was statistically significant increase in excellent, very good, good and fair quality of life and decrease in poor and satisfactory quality of life in intervention group after drug intervention. On the other hand there was no statistically significant change in the study group regarding quality of life. On comparing both control group and intervention group after drug intervention then there was statistically significant difference in quality of life between them with intervention group having better quality of life (Table 3). It was observed that symptoms of the patients improved in 50.67% cases in intervention group and 10.67% cases in control group. The condition of patient remained unchanged in 38.00% cases in intervention group and 73.34% cases in control group. There was worsening of symptoms in 11.34% cases in intervention group and 22.67% cases in control group (Table 4).

## Discussion:

This study was conducted to evaluate the role of LCM as add on treatment modality in dissociative disorders associated with epilepsy. It was observed that there was greater improvement in recovery of symptoms and quality of life in patients of intervention group in which LCM as administered as add on medication. We saw a consistent improvement in the patient's primary symptoms following the addition of lacosamide to their continuing treatment for mood stabilization [1,2]. The research presented here is one of the first to describe a difference in recovery of symptoms and improvement in quality of life in patients with DDs as lacosamide has not been evaluated in the DDs. We chose to use this medication since its safety profile is tolerable and it has demonstrated effectiveness in treating partial-onset seizures [2-6]. Lacosamide has not yet been studied in DD, which means that there is no proof that it could be helpful in this condition. As the FDA approved lacosamide as a monotherapy for partial seizures, it is currently being examined for its cognitive-behavioral effects with the expectation that it won't be linked to memory impairment [7-11]. Although there is a lack of information regarding lacosamide's potential use in treating psychiatric problems, it is plausible that this medication could stop substance-induced mood elevation as well as potentially life-altering occurrences [12- 16]. In an animal model, lacosamide was found to lessen the effects of cocaine on mood. It is important to keep in mind that the primary objective of treating bipolar disorder is to manage manic episodes because doing so keeps the patient from falling into depression [17,21]. Whereas sodium valproate as well as lamotrigine only did so at the maximum dose in the self-stimulation animal's model of mania, lacosamide substantially reduced cocaine-induced intracranial psychological stimulation in rats even at low doses; additionally, at the highest dose, lacosamide raised the self-stimulation thresholds even in the absence of cocaine [21- 24]. This research on animals raises the prospect of using lacosamide as an adjuvant in DD and most likely in drug use disorders as well, whether or not they coexist with DD. In any case, patients with epilepsy with signs of anxiety or depression responded rather well to lacosamide [2-5]. More specifically, a first Spanish research observed a decrease in anxiety/depressive symptoms at the 3- as well as six-month follow-up periods and found lacosamide to eliminate seizures in approximately 55 percent of 31 epileptic patients [22- 26]. In the second research investigation, which was carried out in Ohio, USA, 91 epileptic patients were assessed using the Neurological Disorders Depression Inventory for Epilepsy scale (NDDI-E) [23- 26]. The NDDI-E has a positive likelihood ratio of 0.62, which is fair but not remarkable and does not make the scale appropriate for assessing mental health patients who do not have seizures. After six months, the study showed no discernible change. Nonetheless, there was a noteworthy decrease in depression amongst the 25 patients who had higher-than-average NDDI-E scores (>15). Just 20 patients had a 7-item anxiety test, and the treatment with lacosamide had no discernible effect on anxiety [20-26]. Although dizziness, headaches, vomiting, nasopharyngitis, diplopia, nausea and confusion are the most frequent side effects of lacosamide, a case of psychosis has been reported recently [19-23]. There have been reports of a transient, brief rise in suicide thoughts that disappeared after stopping the medication and switching to an alternative antiepileptic [20-26]. There have also been reports of decreased sexual activity [4-8]. Following the inclusion of lacosamide, we saw no adverse effects in our patient. Our study's findings ought to persuade medical professionals to consider lacosamide for patients with DDs, whether or not they also have epilepsy. Future research may begin as open pilot studies and end with double-blind, randomized controlled trials. Because the primary symptoms of DD took longer to manifest and only partially improved, we are unable to draw the conclusion that lacosamide has promise for treating DD and other trauma-related diseases based on our observations of this study.

## Conclusion:

There was greater improvement in recovery of symptoms and quality of life in patients with DD in which LCM was administered as add on medication.

## Figures and Tables

**Table 1:** Distribution of study participants according to diagnosis of dissociative disorders

	**Irritability**	**Psychosis**	**Anxiety**	**Amnesia**	**Personality change**	**Obsessive Compulsive symptoms**	**Depression**	**Psychogenic non-epileptic seizures**
Study group	54 (36.00)	41 (27.34)	17	7	7	4	4	16
			-11.34	-4.67	-4.67	-2.67	-2.67	-10.67
Control group	56	39	15	9	8	3	5	15
	-37.34	-26	-10	-6	-5.34	-2	-3.34	-10
P value	0.965							

**Table 2:** Quality of life of participants in control group and intervention group before drug intervention

	**Control Group n (%)**	**Intervention Group n (%)**
Excellent	01 (0.67)	01 (0.67)
Very Good	03 (2.00)	04 (2.67)
Good	12 (8.00)	11 (7.34)
Fair	11 (7.34)	12 (8.00)
Satisfactory	44 (29.34)	43 (28.67)
Poor	79 (52.67)	79 ( 52.67)
Total	150 (100)	150 (100)
P value	0.791	

**Table 3:** Quality of life of participants in control group and intervention group after drug intervention

	**Study Group N (%)**	**Intervention Group N (%)**
Excellent	02 (1.34)	11 (7.34)
Very Good	05 (3.34)	13 (8.67)
Good	14 (9.34)	22 (14.67)
Fair	15 (10.00)	21 (14.00)
Satisfactory	43 (28.67)	34 (22.67)
Poor	71( 47.34)	49 (32.67)
Total	150 (100)	150 (100)
P value	<0.001	

**Table 4:** Comparison of relief of symptoms after therapy

	**Intervention group N (%)**	**Control group N (%)**
Improved	76 (50.67)	16 (10.67)
Unchanged	57 (38.00)	110 (73.34)
Worsened	17 (11.34)	34 (22.67)
P value	< 0.001	
